# Adherence Barriers, Patient Satisfaction, and Depression in Albanian Ambulatory Patients

**DOI:** 10.3390/healthcare13141707

**Published:** 2025-07-15

**Authors:** Sonila Qirko, Vasilika Prifti, Emirjona Kicaj, Rudina Cercizaj, Liliana Rogozea

**Affiliations:** 1 Faculty of Medicine, Transilvania University of Brasov, 500036 Brasov, Romania; vasilika.prifti@unitbv.ro (V.P.); emirjona.kicaj@unitbv.ro (E.K.); rudina.cercizaj@unitbv.ro (R.C.); r_liliana@yahoo.com (L.R.); 2 Department of Nursing, Faculty of Health, University of Vlora, 9401 Vlora, Albania

**Keywords:** medication adherence, patient satisfaction, adherence barriers, depression, chronic disease management

## Abstract

**Background:** Medication adherence is essential for managing chronic conditions, while non-adherence remains a widespread issue, leading to poorer health outcomes and higher healthcare costs. This study aimed to identify key adherence barriers, explore their relationship with patient satisfaction, and assess their impact on overall well-being among ambulatory patients in Albania. **Methods:** A cross-sectional study was conducted in three public urban health centers in Vlora, Albania, between November 2024 and January 2025. A total of 80 ambulatory patients were recruited using convenience sampling. Data were collected through face-to-face interviews using validated questionnaires, including the Adherence Barriers Questionnaire (ABQ), the Patient Satisfaction with Nursing Care Quality Questionnaire (PSNCQQ), and the Patient Health Questionnaire (PHQ-9) for depression screening. **Results:** The study included 80 ambulatory patients (mean age 66.7 years; 48.7% female), predominantly diagnosed with diabetes (42.5%) and rheumatic diseases (36.3%). All participants reported at least one adherence barrier, with 92.5% experiencing multiple barriers. The most common were financial burden (91.3%) and fear of side effects (77.5%). A significant positive correlation was found between adherence barriers and depression severity (ρ = 0.518, *p* < 0.0001), while patient satisfaction did not significantly influence adherence barriers (ρ = −0.217, *p* = 0.053) or depression severity (ρ = −0.004, *p* = 0.969). Multiple regression analysis showed that higher depression severity (*p* = 0.0049) was significantly associated with greater adherence barriers, while postgraduate education was associated with fewer barriers (*p* = 0.0175). **Conclusions:** Financial burden, fear of side effects, and psychological distress are key barriers to adherence among Albanian ambulatory patients. Although there are limitations inherent to the cross-sectional design and modest sample size, our findings highlight the potential benefit of routine mental health screening, targeted financial support, and improved patient education on medication management within primary care. These insights may help inform future research and interventions aimed at enhancing adherence and overall well-being. Patient satisfaction did not significantly impact adherence or depression. Targeted interventions focusing on financial support, mental health care, and patient education are needed to improve adherence and patient well-being. These findings underscore the need for integrated mental health and adherence support strategies within routine primary care services.

## 1. Introduction

Medication adherence is a cornerstone of effective chronic disease management. Despite its clinical importance, non-adherence remains a persistent and widespread problem globally, with estimated adherence rates as low as 30–35% in some chronic conditions [1]. Poor adherence is associated with suboptimal clinical outcomes, increased morbidity and mortality, unnecessary hospitalizations, and inflated healthcare costs. Consequently, understanding and addressing the underlying causes of non-adherence has become a priority for both clinicians and public health stakeholders [2].

Adherence is not a unidimensional construct; it is shaped by a wide array of interacting factors. According to the World Health Organization’s Five Dimensions of Adherence framework, these factors span patient-related characteristics (e.g., motivation, beliefs, psychological state), therapy-related complexity (e.g., side effects, dosing frequency), condition-related challenges, socioeconomic context (e.g., financial hardship, education, employment), and the structure and responsiveness of the healthcare system [3,4]. The Adherence Barriers Questionnaire (ABQ) used in this study aligns with key elements of this framework, capturing socioeconomic challenges, therapy-related difficulties, patient beliefs, and health system-related barriers. These diverse influences often overlap, making adherence difficult to predict and address through single-focus interventions.

In low- and middle-income countries (LMICs), socioeconomic barriers such as limited income, lack of insurance coverage, and medication cost are among the most common challenges to adherence [5]. Financial strain not only affects the ability to purchase prescribed medications but can also reduce access to healthcare services more broadly, creating a cycle of untreated illness and poor self-management. Policies promoting medication subsidies, insurance coverage expansion, and improved access to essential medicines are central to alleviating these barriers [6,7].

Another critical yet often overlooked determinant of adherence is psychological well-being [8]. In particular, depressive symptoms have consistently been associated with poor medication adherence across a range of chronic conditions. Depression may impair executive functioning, reduce motivation, and diminish patients’ confidence in their ability to manage their illness—all of which can negatively impact adherence behavior. In this study, we assess depressive symptoms using the Patient Health Questionnaire-9 (PHQ-9) as a validated proxy for psychological well-being. This focus reflects growing recognition of the need to integrate mental health screening and support into chronic disease care, especially in primary care settings.

Patient satisfaction also plays a significant role in shaping adherence behaviors and health outcomes. Satisfied patients are more likely to engage in self-care, trust healthcare professionals, and comply with prescribed treatments. Effective communication, empathy, and continuity of care are among the provider-related factors that influence satisfaction and may indirectly affect adherence [9]. However, evidence on the strength and direction of this relationship remains mixed and context-dependent.

In Albania, few studies have examined the combined influence of psychosocial, economic, and healthcare-related factors on medication adherence in ambulatory populations. This study aims to fill that gap by exploring how perceived barriers to adherence relate to patient satisfaction and depressive symptoms. Grounded in the WHO multidimensional model of adherence, this study seeks to inform the design of integrated, patient-centered interventions that can enhance medication adherence, promote psychological well-being, and ultimately improve the quality of care in primary healthcare.

This study aimed to examine the factors affecting treatment adherence (TA) among outpatients and identify the most significant barriers to adherence, and examine the relationship between adherence barriers, outpatient satisfaction, and overall well-being. By exploring these interactions, the study aimed to provide insights to inform healthcare interventions that enhance adherence, improve patient satisfaction, and ultimately promote better health outcomes.

## 2. Materials and Methods

### 2.1. Study Design

A cross-sectional study was conducted within the context of healthcare services in Albania, aiming to assess treatment adherence, adherence barriers, outpatient satisfaction, and overall well-being among patients.

### 2.2. Study Setting

The study was conducted in three public urban community health centers (HCs) in Vlora, Albania, over a three-month period from November 2024 to January 2025. These centers provide essential healthcare services to an urban population of approximately 144,000 residents, offering preventive care, chronic disease management, maternal and child health services, and treatment for minor emergencies.

The selection of these three centers was based on predefined criteria, including high patient flow, geographic distribution within the city, and the availability of chronic care services. These centers were chosen due to their representativeness of the urban primary healthcare setting in Vlora, ensuring diversity in patient demographics and health conditions. The inclusion of centers from different urban neighborhoods allowed for the capture of a more comprehensive picture of adherence behaviors and patient satisfaction across varying socio-economic strata.

Health centers located in rural areas were excluded from the study to maintain contextual consistency and focus on urban-specific adherence challenges. Rural areas often differ in healthcare infrastructure, access, and patient-provider dynamics, which could introduce confounding variables not aligned with the primary objectives of the study. By focusing solely on urban centers, the study aimed to evaluate adherence-related issues in a setting characterized by higher patient density, more specialized healthcare staff, and relatively greater access to medical resources.

### 2.3. Participants

A convenience sampling approach was used to recruit patients receiving ambulatory care for routine follow-up or chronic disease management at three health centers (HCs) in the Vlora district. This method was selected due to the practical constraints of real-time recruitment in outpatient settings. While appropriate for the exploratory design of the study, we acknowledge that it may introduce selection bias and limit the generalizability of the results. Eighty-three patients were initially enrolled, but three surveys with missing responses on core variables were excluded, resulting in a final analytic sample of 80 complete cases. Inclusion criteria required participants to be 18 years or older, fluent in Albanian, cognitively alert, physically capable of answering survey questions, and willing to participate. Only patients with chronic medical conditions (e.g., diabetes, cardiovascular disease, rheumatic disease) were included in this study. Individuals with known psychiatric diagnoses, including clinically diagnosed depression or anxiety disorders, were excluded to reduce potential confounding in the assessment of depressive symptoms and treatment adherence. Exclusion criteria included individuals who were unable to provide informed consent, or had significant communication barriers, such as non-Albanian speakers or individuals with hearing impairments without support. Patients with a current diagnosis of depression or other psychiatric conditions under treatment were excluded from the study. Only depressive symptoms as screened by the PHQ-9 instrument were considered for analysis, without any formal psychiatric diagnosis being part of the inclusion criteria.

Data collection was conducted through face-to-face interviews by four trained researchers immediately following patients’ medical visits. No open-ended or qualitative interview guide was used. All interviews were strictly structured, based solely on three previously validated instruments (ABQ, PSNCQQ, PHQ-9). No additional themes outside the scope of these questionnaires were explored, and no qualitative analysis was performed. Recruitment was carried out using convenience sampling. Patients were approached in the waiting area after completing their medical consultation. Researchers introduced themselves, briefly explained the purpose of the study, and invited them to participate voluntarily.

Those who expressed initial interest were given a more detailed explanation of the study, including information about confidentiality, anonymity, and their right to withdraw at any time. Written informed consent was obtained prior to participation. If patients declined to participate, the most commonly cited reasons were lack of time or disinterest.

The interviews were conducted in a private area within the health center to ensure comfort and confidentiality. Each interview lasted approximately thirteen minutes and was carried out by one of the trained researchers, all of whom were medical or public health professionals with prior experience in data collection and patient communication. All data collectors received structured training from the principal investigator prior to study initiation. The training covered the standardized administration of the questionnaires, ethical considerations (e.g., informed consent, confidentiality), and strategies to minimize interviewer bias. Role-play simulations and supervised pilot testing were used to ensure consistency across interviewers. Socio-economic status (SES) was categorized based on participants’ self-reported monthly household income. These income ranges were grouped into low, moderate, and high categories in accordance with thresholds from national benchmarks established by the Albanian Institute of Statistics (INSTAT), reflecting income distribution in urban areas [10]. Incomplete surveys and participants who did not provide full consent were excluded from the final analysis to ensure data quality and integrity.

### 2.4. Data Sources/Measurement

#### Instrument Used

Several validated questionnaires were utilized in this study, including the Adherence Barriers Questionnaire (ABQ) [11]. It is important to note that this study did not directly assess medication adherence behavior (e.g., self-reported adherence frequency or refill records). Instead, it focused on identifying perceived barriers to adherence using the Adherence Barriers Questionnaire (ABQ), which captures obstacles that may influence adherence but does not quantify adherence itself. The other two questionnaires, Patient Satisfaction with Nursing Care Quality Questionnaire (PSNCQQ) [12] and the Patient Health Questionnaire-9 (PHQ-9), a widely recognized tool for assessing depressive symptoms, both have been previously validated and adapted for outpatient settings in Albania ensuring linguistic and cultural appropriateness for this study context. The PSNCQQ is a validated tool designed to assess patient satisfaction with various dimensions of nursing care. It includes 19 items covering areas such as responsiveness, communication, emotional support, and professionalism. Each item is rated on a 5-point Likert scale ranging from “poor” to “excellent.” For this study, responses were dichotomized into two groups: excellent/very good vs. fair/poor, following established thresholds in health services research. In this study, PHQ-9 had Cronbach’s α = 0.86 and PSNCQQ had α = 0.91 [13].

The PHQ-9 consists of nine items scored on a scale from 0 (not at all) to 3 (nearly every day), with total scores ranging from 0 to 27. Depression severity was categorized as follows: 0–4 (no/minimal depression), 5–9 (mild), 10–14 (moderate), 15–19 (moderately severe), and 20–27 (severe depression). Patient satisfaction with nursing care was dichotomized into two groups: excellent/very good vs. poor/fair. This dichotomization was based on a commonly used threshold in health services research to distinguish between overall positive and negative perceptions of care. The decision to group “excellent” and “very good” together reflects a high level of satisfaction likely to influence adherence, whereas “fair” and “poor” responses suggest dissatisfaction that may impact patient behavior and perception. This approach also facilitated more meaningful statistical comparisons between groups, particularly given the sample size.

The ABQ consists of 16 items assessing various categories of barriers, including patient-related, therapy-related, and socioeconomic factors. Each item is rated on a 4-point Likert scale ranging from “strongly agree” to “strongly disagree,” with certain items reverse-coded to maintain directional consistency. Scores range from 16 to 64. A total ABQ score of >25, or an average item score above 2, was used as the threshold for clinically significant adherence barriers, based on prior validation studies, psychometric guidance, and internal pilot testing [11]. To evaluate adherence barriers, individual item-specific scores and a total ABQ score were calculated. Patients were classified as experiencing adherence barriers if their average item score exceeded 2, or if at least one subscale item received a score of 4. The prevalence of each barrier was determined by reporting the number and proportion of affected patients. Responses were categorized into “affected” and “not affected” groups, where patients were considered affected if they responded with a score greater than 2 (i.e., “slightly disagree” or “strongly disagree” after reverse coding). The total ABQ score was derived by summing all individual item scores.

### 2.5. Psychometric Properties of the ABQ

#### 2.5.1. Validity and Reliability Analysis

An analysis of validity, reliability, and factor structure was conducted for the questionnaire to assess its overall measurement properties and ensure its robustness.

#### 2.5.2. Translation and Content Validity

The ABQ was translated into Albanian using a forward–backward translation process to ensure linguistic and conceptual equivalence. A team of five bilingual experts, including medical and linguistic professionals, reviewed and refined the wording of the 16 items. Back-translation by two trained linguists confirmed consistency with the original scale, with minor adjustments made to align with cultural context. The Content Validity Index (CVI) was used to assess item validity, and a pilot study with 20 patients (October–November 2024) helped identify unclear items, leading to minor refinements.

#### 2.5.3. Construct Validity

Factor analysis of the Albanian version of the scale yielded a single-factor structure, with factor loadings ranging from 0.721 to 0.938.

### 2.6. Internal Consistency

The ABQ demonstrated strong internal consistency, with item correlation coefficients ranging from 0.70 to 0.91. Cronbach’s α = 0.872, indicating high reliability.

### 2.7. Test–Retest Reliability

To evaluate stability, the questionnaire was re-administered to 20 patients three weeks later, yielding consistent results, confirming the scale’s reliability over time. The Albanian version of the ABQ was found to have excellent psychometric properties, aligning closely with the original scale.

### 2.8. Bias Management

Various measures were taken to reduce potential bias in this study. Clearly defined inclusion and exclusion criteria ensured a relevant and suitable sample. The ABQ underwent a thorough adaptation and validation process, including forward–backward translation, expert review, and pilot testing, to maintain cultural and contextual accuracy.

To minimize variability, trained researchers conducted standardized face-to-face interviews during data collection. A pilot study was conducted to identify and resolve any ambiguities in the questionnaires, enhancing clarity and reducing response bias. Ethical guidelines, such as obtaining informed consent, were strictly followed to ensure voluntary and impartial participation. Additionally, appropriate statistical methods were employed to enhance the robustness and reliability of the findings.

### 2.9. Study Size

Based on G*Power 3.1.9.7 analysis, a sample size of 64 was required to detect a moderate effect size (Cohen’s d = 0.5) with 80% power (α = 0.05, two-sided test). To account for a 20% non-response rate, the study included 80 ambulatory patients, ensuring statistical reliability while addressing potential missing data and variability.

### 2.10. Statistical Methods

Data collected during the study were analyzed using the IBM Statistical Package for the Social Sciences (SPSS), version 25.0 (released 2017; IBM Corp., Armonk, NY, USA). The Shapiro–Wilk test was used to assess the normality of the distribution of continuous variables. Descriptive statistics, including means and standard deviations (SD) for continuous variables, and percentages for categorical variables, were used to summarize the data. Categorical variables were analyzed using chi-square test. The nonparametric Spearman correlation was used to assess the relationship between adherence barriers with patient satisfaction and depression. A multiple linear regression analysis was conducted to identify the sociodemographic and psychological factors associated with the total adherence barriers score (Sum ABQ). The dependent variable was the total score derived from the 16-item Adherence Barriers Questionnaire (ABQ), treated as a continuous outcome. Independent variables included depression severity (PHQ-9 total score), patient satisfaction, gender, age, education level, marital status, employment status, socio-economic status, and healthcare center. The two-tailed level of statistical significance was set at *p* < 0.05 for all comparisons.

### 2.11. Ethical Considerations

All ethical guidelines, including those specified in the Helsinki Declaration, were strictly followed in this study. Approval was obtained from the instrument’s developer. Before data collection, the research protocol was reviewed and approved by the Scientific Ethics Committee of the University “Ismail Qemali” (Approval No. 113/1 dated 25 April 2024). Participation was entirely voluntary, with patients providing written informed consent and having the right to withdraw at any time. All ethical issues according to the Helsinki declaration were strictly followed.

## 3. Results

Socio-demographical characteristics of the participants (N = 80) are presented in Table 1. The mean age of the sample was 66.7 ± 11.7 years old with a range 42 to 89 years, and 55% were aged between 50 and 59 years. In this study, diabetes was the most prevalent condition, affecting 34 patients (42.5%), followed by rheumatic diseases, reported in 29 cases (36.3%). Gastrointestinal, pulmonary, and renal diseases were each observed in 13 cases (16.3%). Notably, many patients had more than one chronic condition, highlighting a high burden of multi morbidity.

Out of a total of 80 patients, 4 (5%) of them had a score > 25 ABQ points. Despite adherence barriers, those four patients were satisfied with nursing care. The results indicate that gender and education level were significantly associated with adherence barriers, with women and those with lower education levels experiencing higher adherence barriers. A significant association was observed between gender and ABQ score categories (*p* = 0.036), indicating that all individuals with >25 ABQ points were female (100.0%), whereas the ≤25 ABQ points group included both males (53.9%) and females (46.1%). Additionally, a statistically significant relationship was found between education level and ABQ scores (*p* < 0.001). Among individuals with ≥25 ABQ points, 50.0% had completed only secondary education, while 25.0% had a high school or university degree, and none had a postgraduate degree. Other demographic variables, such as age, marital status, socioeconomic status, employment, and healthcare center, did not demonstrate statistically significant differences between the high and low adherence barrier groups.

**Figure 1:**
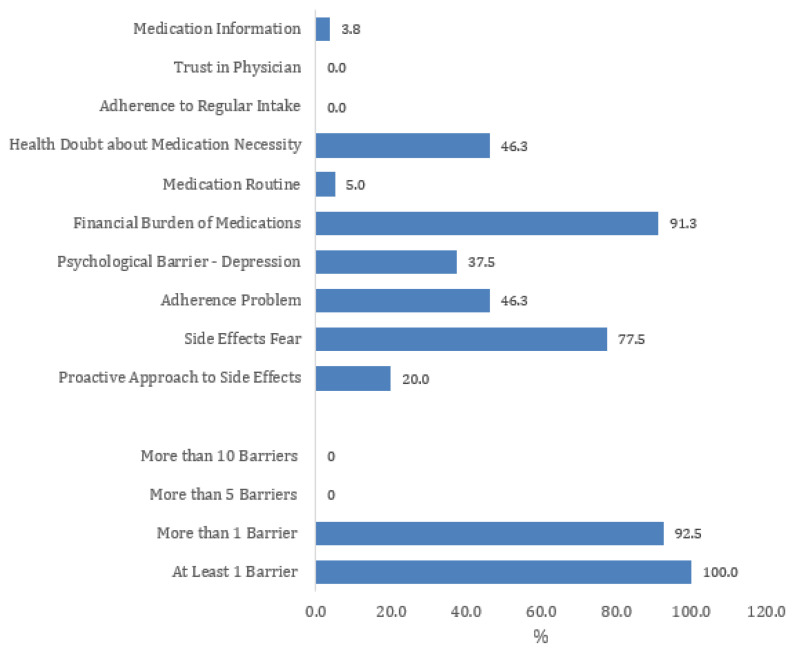
Percentage of patients impacted by adherence barriers according to the ABQ assessment.

The analysis of adherence barriers revealed that all patients (100%) experienced at least one barrier, with 92.5% reporting more than one barrier, underscoring the complexity of adherence challenges (Figure 1). Among the most prevalent barriers, financial burden of medications (91.3%) emerged as the most significant obstacle, followed by fear of side effects (77.5%), indicating that economic constraints and concerns about adverse effects play a critical role in medication adherence. Additionally, 46.3% of patients reported adherence problems, and an equal proportion expressed doubts about the necessity of their medication, suggesting that nearly half of the sample struggles with maintaining their treatment regimen or questions its importance. Psychological factors also influenced adherence, with 37.5% of patients identifying depression as a barrier, highlighting the role of mental health in treatment compliance. However, only 20.0% reported taking a proactive approach to managing side effects, suggesting that most patients may not actively seek strategies to mitigate medication-related concerns.

**Figure 2:**
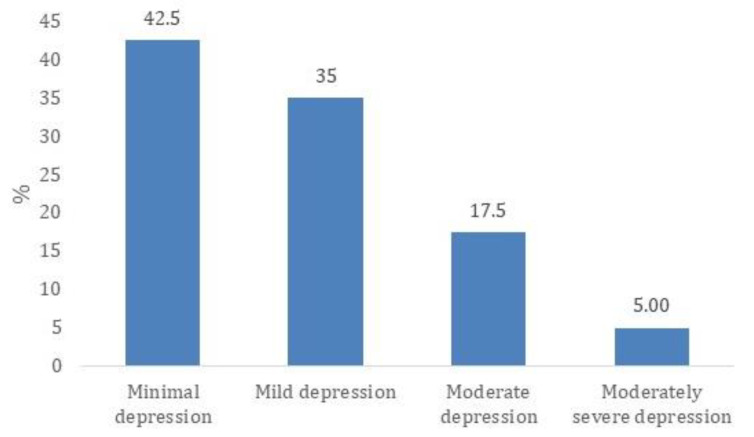
Distribution of depression severity levels based on PHQ-9 scores.

The majority of patients, 34 out of 80 (42.5%), fall into the minimal depression category, suggesting that nearly half of the sample experiences little to no depressive symptoms (Figure 2). Another 28 patients (35.0%) exhibit mild depression, indicating that more than one-third have some symptoms but may not require clinical intervention.

A smaller portion, 14 patients (17.5%), are classified as having moderate depression, where symptoms could start to interfere with daily life. Meanwhile, only 4 patients (5.0%) fall into the moderately severe depression category, suggesting that a very small percentage experience significant impairment that may require medical attention.

The majority of patients (76 out of 80; 95.0%) had ABQ scores ≤ 25, while only 4 patients (5.0%) scored ≥ 25 ABQ points. Depression severity, as measured by PHQ-9, showed no significant association with ABQ scores (χ^2^ = 3.17, df = 3, *p* = 0.365). Among those with ≥25 ABQ points, 3 (75.0%) had mild depression, and 1 (25.0%) had minimal depression, with no cases of moderate or moderately severe depression. Similarly, there was no significant association between depression severity and patient satisfaction (χ^2^ = 1.65, df = 3, *p* = 0.646). Among those with mild depression, 26 patients (33.8%) were satisfied, while 2 (66.7%) were dissatisfied. In the minimal depression category, 33 patients (42.9%) reported satisfaction, and 1 (33.3%) was dissatisfied. All patients with moderate and moderately severe depression were satisfied, with no cases of dissatisfaction in these groups. These findings indicate no meaningful relationship between ABQ scores, depression severity, and dissatisfaction in this sample. While no statistically significant associations were observed between ABQ score categories and PHQ-9 depression severity groups using chi-square tests, continuous variable analysis demonstrated a moderate-to-strong positive association. Specifically, Spearman’s correlation showed a significant relationship between ABQ scores and PHQ-9 scores (ρ = 0.554, *p* < 0.0001), and multiple linear regression confirmed that higher depression severity significantly predicted higher adherence barriers (B = 0.2991, *p* = 0.0049). This highlights that while categorical group comparisons did not yield significance, the continuous analysis reveals an underlying association between depressive symptoms and perceived treatment barriers.

The correlation analysis showed no significant association between satisfaction and PHQ-9 scores (ρ = −0.004, *p* = 0.969) or between satisfaction and ABQ score (ρ = −0.217, *p* = 0.053) indicating that satisfaction is not meaningfully influenced by depression severity or adherence barriers.

**Figure 3:**
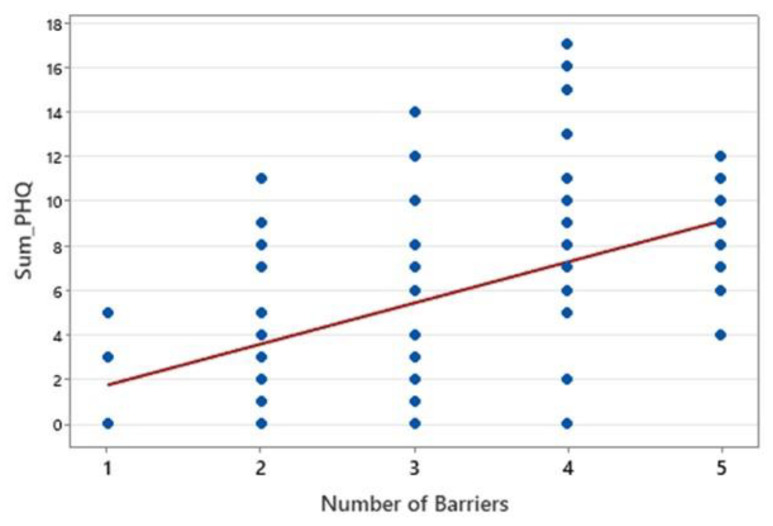
Correlation between number of barriers and PHQ score.

A significant positive correlation was found between the number of barriers and PHQ-9 depression scores (ρ = 0.518, *p* < 0.0001), indicating a moderate association (Figure 3). The 95% confidence interval (0.336 to 0.662) confirmed that this relationship ranges from weak to moderate, suggesting that more barriers are linked to higher depressive symptoms. These findings highlight the impact of adherence barriers on mental health, emphasizing the need for further research into their specific role in psychological distress.

**Figure 4:**
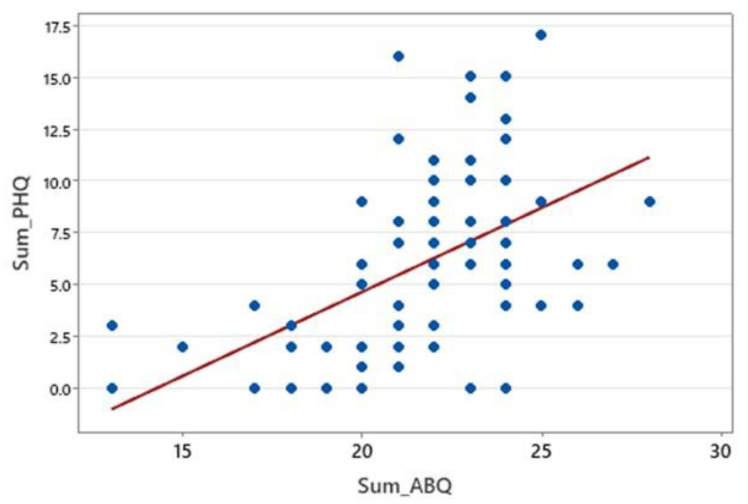
Correlation between ABQ score and PHQ score.

A moderate positive correlation was found between ABQ scores and PHQ-9 depression scores (ρ = 0.554, *p* < 0.0001), indicating that higher adherence barriers were associated with greater depressive symptoms (Figure 4). These findings suggest a significant link between adherence difficulties and depression severity.

Table 2 presents the results of the multiple linear regression analysis (Adjusted R^2^ = 0.228; F(15,64) = 2.61, *p* = 0.0043), assessing associations between participant characteristics and total adherence barriers, while adjusting for potential confounders.

Among the predictors, higher depression severity (PHQ-9 score) was significantly associated with an increase in adherence barriers (B = 0.25, *p* = 0.013), suggesting that each additional point in depression score predicted a 0.25-point increase in the Sum ABQ score. In contrast, individuals with a postgraduate education reported significantly lower adherence barriers compared to those with other education levels (B = −6.61, *p* = 0.017).

None of the other sociodemographic factors—including gender, age, marital status, employment status, socioeconomic status, or satisfaction with care—demonstrated a statistically significant association with the total ABQ score. Similarly, no significant differences in adherence barriers were observed across the three participating healthcare centers. These findings highlight the prominent role of psychological distress—particularly depression—as a key driver of perceived treatment barriers, while educational level may serve as a protective factor. Other demographic characteristics appeared to have limited influence in this population.

## 4. Discussion

Medication adherence is influenced by both patient-centered and provider-centered factors. Patients with greater awareness of their medical condition are more likely to adhere to prescribed treatments, while those with negative perceptions of their illness tend to exhibit lower adherence rates [14]. Research has also shown that depression and psychological distress contribute to lower adherence, as they impact motivation and self-efficacy in treatment management [15]. Non-adherence represents a significant public health concern, contributing to higher treatment costs, increased disease burden, and poorer patient outcomes [16].

This study examined treatment adherence barriers, patient satisfaction, and their impact on well-being among ambulatory patients in Albania. The findings highlight financial burden, fear of side effects, and doubts about medication necessity as the most significant barriers, with depression and psychological distress strongly linked to increased perceived barriers.

Financial burden emerged as the most frequently cited obstacle, affecting over 90% of participants. This aligns with other studies in low- and middle-income countries where high out-of-pocket costs are a major cause of medication non-adherence [17,18,19]. In Albania, where out-of-pocket payments account for approximately half of total health expenditures, financial hardship can directly impede access to necessary medications [20]. This economic strain may also act as a chronic stressor, increasing psychological distress and depressive symptoms, which in turn further undermine patients’ motivation and capacity to adhere to treatment [20]. Patients under financial pressure may ration medications, skip doses, or delay refills, creating a cycle of poor disease control and rising costs.

Fear of side effects was the second most commonly reported barrier, cited by 77.5% of participants. Previous research shows that patients who experience or anticipate adverse reactions often stop or adjust medications without medical guidance [21,22]. This emphasizes the need for accessible counseling and clear communication about managing side effects. Interestingly, trust in physicians was not identified as a major barrier in this sample. While some healthcare systems report poor physician–patient relationships as a factor in non-adherence [23], the Albanian system reflects a traditionally hierarchical structure where strong physician authority may limit open dialog about treatment concerns, costs, or emotional distress. This cultural dynamic could discourage patients from expressing fears or asking for help managing barriers such as depression or medication side effects [24].

Depression was a significant psychological factor associated with increased adherence barriers. The positive correlation between adherence barriers and PHQ-9 scores in our study supports existing evidence that depression impairs self-efficacy, decision-making, and motivation to manage chronic conditions [25,26,27]. This highlights the importance of integrating mental health support into routine primary care, particularly in societies where mental health remains stigmatized and under-addressed [28,29].

Taken together, these findings align with the WHO’s Five Dimensions of Adherence framework: financial burden reflects the socioeconomic domain; fear of side effects relates to therapy-related factors; depression illustrates the patient-related dimension; and the limited role of patient satisfaction suggests opportunities to strengthen the health system’s responsiveness and patient–provider communication [3,4].

While postgraduate education appeared protective against perceived barriers, other sociodemographic factors such as age, gender, marital status, employment status, and socioeconomic status did not show significant associations in this sample. This suggests that, in this context, financial and psychological challenges may outweigh other demographic influences [30,31,32,33].

These findings underscore the need for multifaceted interventions that address both structural and patient-level barriers. Strategies should include expanding financial assistance or reimbursement schemes [34,35], offering affordable generic alternatives, and providing clear patient education about medication necessity and side-effect management [36]. Importantly, mental health screening and basic psychosocial support should be routinely integrated into primary care settings to address the psychological burden that complicates adherence [37].

Future research should explore how patient–provider communication, cultural factors, and community-level stigma shape adherence behaviors and openness to mental health services [32]. Longitudinal studies would help clarify the causal pathways between financial hardship, depression, and adherence over time.

Addressing adherence barriers in Albania’s ambulatory care settings will require an integrated approach that combines financial relief, strengthened patient education, and routine mental health support—all embedded within a more patient-centered, responsive primary care system [38,39].

### Study Limitations and Future Research

This study has certain limitations. First, the study population was drawn exclusively from three urban health centers in Vlora, Albania. However, the small sample size may limit generalizability beyond the urban Albanian population.

This limitation should be considered when interpreting the applicability of the results to broader national or regional contexts. The relatively small sample size (n = 80) may have restricted the detection of smaller effect sizes or variations between subgroups. Additionally, the cross-sectional design limits the ability to establish causal links between adherence barriers, depression, and patient satisfaction. Furthermore, adherence was assessed using self-reported questionnaires, which could introduce recall bias or social desirability bias. While the PHQ-9 was used to assess depressive symptoms, no clinical diagnostic evaluation or formal mental health intervention was provided to participants. The tool served as a screening instrument only, and patients who scored high on depressive symptoms were not followed up with psychiatric referrals or counseling as part of the study. This limitation is acknowledged, as it may impact the clinical interpretation of depression-related findings.

Despite these limitations, the study is strengthened by its use of validated instruments, its multidimensional approach encompassing psychological, economic, and service-related factors, and its focus on a real-world ambulatory care population.

Future studies should prioritize longitudinal research to track changes in adherence behaviors over time and whether interventions targeting financial and psychological barriers lead to measurable improvements in adherence and patient well-being. Expanding the study to include larger and more diverse patient populations will further enhance the generalizability and applicability of the findings.

## 5. Conclusions

This study highlights the complex interplay between adherence barriers, psychological well-being, and patient satisfaction among ambulatory patients with chronic conditions in Albania. Depression severity emerged as a key predictor of perceived barriers, suggesting that routine mental health screening and basic psychological support within primary care may be beneficial. The protective effect of higher education suggests that improving health literacy may empower patients to manage their treatments more effectively.

These findings reinforce the need for an integrated, multidisciplinary approach that combines targeted financial assistance, structured patient education on medication necessity and side-effect management, and accessible mental health services. For example, routine PHQ-9 depression screening could be embedded into primary care visits, and nurses or primary care teams may benefit from training to provide basic counseling or timely referrals to community mental health services.

Policymakers and healthcare providers could also consider expanding insurance coverage, implement financial aid or reimbursement schemes for essential medications, and encourage the use of affordable generic drugs to reduce out-of-pocket costs. Addressing these structural barriers may be critical for improving equitable access to treatment.

Future research should examine the impact of social support and health literacy on adherence, as well as test the long-term effectiveness of multifaceted interventions that combine financial relief, patient education, and mental health integration.

In conclusion, improving medication adherence may require coordinated actions that go beyond patient satisfaction. Policies and clinical practices could align to reduce economic strain, normalize mental health care within chronic disease management, and equip patients with the knowledge and support they need to adhere to treatment and achieve better health outcomes.

## Figures and Tables

**Table 1:** Sociodemographic characteristics of all patients based on high and low adherence barriers.

Variables	Total (n = 80)	≤25 ABQ Points (n = 76)	>25 ABQ Points (n = 4)	*p*-Value
Age group				0.603
40–49	23 (28.7)	22 (28.9)	1 (25.0)	
50–59	44 (55.0)	41 (53.9)	3 (75.0)	
60–89	13 (16.3)	13 (17.2)		
Gender				0.036
Female	39 (48.7)	35 (46.1)	4 (100.0)	
Male	41 (51.2)	41 (53.9)	0	
Marital status				0.619
Divorced	4 (5.0)	4 (5.3)	0	
Married	67 (83.7)	64 (84.2)	3 (75.0)	
Widow/er	9 (11.3)	8 (10.5)	1 (25.0)	
Education level				<0.001
Secondary	43 (53.7)	0	2 (50.0)	
High school	22 (27.5)	21 (27.6)	1 (25.0)	
University	14 (17.5)	13 (17.1)	1 (25.0)	
Postgraduate	1 (1.3)	1 (1.3)	0	
Socio-economic status				0.254
Low	15 (18.8)	13 (17.1)	0	
Moderate	63 (78.7)	61 (80.3)	2 (50.0)	
High	2 (2.5)	2 (2.6)	2 (50.0)	
Employment status				0.677
Unemployed	11 (13.8)	11 (14.5)	0	
Employed	22 (27.5)	21 (27.6)	1 (25.0)	
Retired	47 (58.7)	44 (57.9)	3 (75.0)	
Healthcare Center				0.860
HC no.1	35 (43.7)	33 (43.4)	2 (50.0)	
HC no.3	30 (37.5)	29 (38.2)	1 (25.0)	
HC no.5	15 (18.8)	14 (18.4)	1 (25.0)	

ABQ ≤ 25 points—no adherence barrier. ABQ > 25 points—adherence barrier. *p* ≤ 0.05 is significant.

**Table 2:** Multiple linear regression analysis of predictors of total adherence barrier score.

Variable	Coefficient	Std.Error	t-Value	*p*-Value	CI Lower	CI Upper
Intercept	26.1527	4.9974	5.2333	0.0000	16.1564	36.148
Gender/Male	−0.0528	0.6448	−0.082	0.9350	−1.3427	1.2370
Age	−0.0893	0.0790	−1.129	0.2631	−0.2473	0.0688
Age group/50–59	2.7139	1.3872	1.9563	0.0551	−0.0610	5.4887
Age group/60–89	2.5795	2.3295	1.1073	0.2726	−2.0802	7.2392
High Education	−0.1514	0.8997	−0.168	0.8669	−1.9511	1.6484
Postgraduate	−6.6375	2.7158	−2.444	0.0175	−12.070	−1.2050
Secondary Education	−0.2372	0.9143	−0.259	0.7962	−2.0662	1.5917
Married	−0.3343	1.3407	−0.243	0.8040	−3.0160	2.3475
Widow/er	0.4830	1.5590	0.3098	0.7578	−2.6354	3.6015
Employed	−0.4166	1.3104	−0.317	0.7517	−3.0378	2.2046
Retired	0.0284	1.2005	0.0237	0.9812	−2.3730	2.4298
SES/Moderate	−0.0962	2.0129	−0.047	0.9620	−4.1227	3.9303
SES/Low	0.2221	2.1004	0.1057	0.9161	−3.9793	4.4235
Health Center/nr. 3	0.6720	0.7149	0.9400	0.3510	−0.7581	2.1021
Health Center/nr. 5	−0.4713	0.8317	−0.566	0.5731	−2.1349	1.1924
PHQ9_score	0.2991	0.1020	2.9233	0.0049	0.0945	0.504
Satisfaction	−1.8985	1.6761	−1.132	0.2618	−5.2511	1.4542

CI 95% confidence interval. *p* ≤ 0.05 is significant.

## Data Availability

The raw data supporting the conclusions of this article will be made available by the authors on request.

## Abbreviations

The following abbreviations are used in this manuscript:

ABQ	Adherence Barriers Questionnaire
PHQ-9	Patients Health Questionnaire
PSNCQQ	Patients Satisfaction with Nursing Care Quality
AB	Adherence Barriers
SES	Socioeconomic Status
